# Roles of Inflammasome in Cigarette Smoke-Related Diseases and Physiopathological Disorders: Mechanisms and Therapeutic Opportunities

**DOI:** 10.3389/fimmu.2021.720049

**Published:** 2021-07-21

**Authors:** Yiming Ma, Yingjiao Long, Yan Chen

**Affiliations:** Department of Pulmonary and Critical Care Medicine, The Second Xiangya Hospital, Central South University, Changsha, China

**Keywords:** inflammasome, cigarette smoke, NLRP3, ROS, innate immune

## Abstract

Cigarette smoke damages a wide range of immunological functions, including innate and adaptive immune responses. Emerging literature demonstrates that inflammasome constitutes an essential component in innate immune response. In this review, we focus on the cumulative mechanisms of inflammasome in cigarette smoke-related diseases and physiopathological disorders, and summarize potential therapeutic opportunities targeting inflammasome. This review suggests that inflammasomes (NLRP3, NLRP6, NLRP12 and AIM2) are involved in the pathogenesis of several cigarette smoke-related diseases (including COPD, ALI, atherosclerosis, kidney injury, bladder dysfunction, and oral leukoplakia) and physiopathological disorders (macrophage dysfunction, endothelial barrier dysfunction, podocyte injury, and ubiquitin-mediated proteasomal processing). MyD88/NF-κB, HMGB1, production of ROS, endoplasmic reticulum stress and mitochondrial dysfunction, and Ca^2+^ influx are potentially involved in cigarette smoke induced-inflammasome activation. Strategies targeting ROS/NLRP3 inflammasome axis are most widely investigated and show potential therapeutic effects.

## Introduction

Cigarette smoke remains to be a threat to the health of the world’s population, and a global survey has indicated that prevalence of daily tobacco smoking in the population older than 15 years is 31.1% for men and 6.2% for women (1). Previous studies have demonstrated that cigarette smoke is a major risk factor for occurrence and progress of diseases involving multiple systems throughout the body, such as respiratory system (2–4), cardiovascular system (4, 5), and nervous system (6, 7). Moreover, chronic cigarette smoke inhalation damages a wide range of immunological functions, including innate and adaptive immune responses (8).

Inflammasomes are multiprotein signaling platforms mediating inflammatory responses and coordinating antimicrobial host defenses (9–12). Assembly of an inflammasome complex requires cytosolic sensing of pathogen-associated molecular patterns or danger-associated molecular patterns by a nucleotide-binding domain and leucine-rich repeat receptor (NLR) or absent in melanoma (AIM) 2-like receptor (13). Inflammasomes could activate proinflammatory caspase-1, and caspase-11 (in mice)/caspase-4 and caspase-5 (in humans), which further leads to maturation of interleukins 1β and 18 (IL-1β and IL-18) through proteolytic cleavage of pro-IL-1β and pro-IL-18 (14–16); besides, activated caspase-1, and caspase-11 (in mice)/caspase-4 and caspase-5 (in humans) also cleave and activate Gasdermin D (GSDMD), which induces a type of cell death called pyroptosis (15, 17, 18). The NLRP3 inflammasome has been under intensive study due to its extensive connection with a variety of human diseases and a two-signal model has been proposed for NLRP3 inflammasome activation; in this model, the first signal (priming) is provided by microbial or endogenous molecules that induce NLRP3 and pro-IL-1β expression through activation of NF-κB; the second signal (activation) is triggered by K+ efflux, Ca^2+^ signaling, reactive oxygen species (ROS), mitochondrial dysfunction, and lysosomal rupture (19). In clinical practice, VX-765, an inhibitor that directly targets inflammasome downstream cytokines, is able to block the hypersensitive response to an inflammatory stimulus in monocytes from familial cold autoinflammatory syndrome patients, which provides a novel alternative in treating immune-associated diseases (20). In this review, we focus on the cumulative mechanisms of inflammasome in cigarette smoke-related diseases and physiopathological disorders, and summarize potential therapeutic opportunities targeting inflammasome.

## Roles of Inflammasome in Cigarette Smoke-Related Diseases

### Inflammasome in Cigarette Smoke-Related Lung Diseases

#### Stable Chronic Obstructive Pulmonary Disease (COPD)

Roles of inflammasome in cigarette smoke-related diseases and physiopathological disorders are summarized in **Table 1**. The role of inflammasome in stable COPD has been widely investigated both *in vitro* and *in vivo* models, while the conclusion remains contradictory. In vitro, Mortaz et al. (38) found that cigarette smoke could increase the expression of caspase-1 and IL-1β in human alveolar epithelial cells, suggesting the potential role of inflammasome signaling in the pathogenesis of COPD. Thereafter, more studies further demonstrated cigarette smoke was able to activate NLRP3 inflammasome in human bronchial and alveolar epithelial cells, and the inflammasome activation was able to further increase the release of inflammatory cytokines (including IL-1β and IL-18) (21, 22, 26, 27). Particularly, Mahalanobish et al. (21) reported that the activation of NLRP3 inflammasome in alveolar epithelial might result from cigarette smoke induced endoplasmic reticulum (ER) stress and mitochondrial dysfunctions. Wang et al. (27) found that cigarette smoke-induced inflammasome activation was triggered by oxidative stress injury and Ca^2+^ influx in human bronchial and alveolar epithelial cells. Besides the activation of NLRP3 inflammasome, Singh et al. (31) observed that increase of NLRP10 and NLRP12 proteins in human alveolar type II epithelial cells challenged by cigarette smoke extract. Furthermore, Kaur et al. (32) proved that NLRP10 knockdown rescued cigarette smoke extract induced inflammatory responses in human alveolar type II epithelial cells, which might serve as an effective therapeutic target of COPD.

**Table 1:** Roles of inflammasome in cigarette smoke-related diseases and physiopathological disorders.

Reference	Year	Disease/disorder	Sample/Subjects	Effect of cigarette smoke on inflammasome
Mahalanobish et al. (21)	2020	COPD	Mouse lung tissue and BALF, human alveolar epithelial cells	Induce endoplasmic reticulum stress and mitochondrial dysfunctions, and further activate NLRP3 inflammasome
Rumora et al. (22)	2020	COPD	Human bronchial epithelial cells, monocyte-derived macrophages, and THP-1 cells	Increase NLRP3 and IL-1β
Rumora et al. (23)	2021	AECOPD	Human bronchial epithelial cells, monocyte-derived macrophages, and THP-1 cells	Increase NLRP3 and IL-1β
Ji et al. (24)	2020	AECOPD	Rat lung tissue and BALF, human bronchial epithelial cells	Increase caspase-1, NLRP3, IL-1β and IL-18
Ozretic et al. (25)	2019	COPD	Human peripheral blood mononuclear and lung fibroblast cells	NLRP1 rs12150220 coding polymorphisms are associated with COPD disease severity
Nachmias et al. (26)	2019	COPD/AECOPD	Human alveolar epithelial cells	Increase NLRP3 and IL-1β
Wang et al. (27)	2019	COPD	Human bronchial and alveolar epithelial cells	Induce oxidative stress injury, promote Ca^2+^ influx, and increase caspase-1, NLRP3, IL-1β and IL-18
Colarusso et al. (28)	2019	AECOPD	Human peripheral blood mononuclear cells	Increase AIM2/caspase-1/caspase-4 in IL-1α-induced TGF-β release
Cao et al. (29)	2018	COPD	Mouse lung tissue and BALF	Induce ROS production and increase NLRP3, cleaved-IL-1β and cleaved-caspase-1
Wang et al. (30)	2018	AECOPD	Human peripheral blood mononuclear cells, bronchial tissues, serum and BALF	Increase NLRP3, caspase -1, ASC, IL-18 and IL-1β
Singh et al. (31)	2018	COPD	Human alveolar epithelial cells	Increase NLRP10, NLRP12, caspase-1, IL-1β, and IL-18
Kaur et al. (32)	2018	COPD	Mouse lung tissue, human alveolar epithelial cells	Increase NLRP10, caspase-1, IL-1β, and IL-18
Faner et al. (33)	2016	COPD/AECOPD	Human lung tissue of stable COPD, human sputum and plasma of AECOPD	Stable COPD: NLRP3 inflammasome is primed, but not activated; both caspase-1 and ASC were mostly inactive
Yang et al. (34)	2016	COPD	Mouse BALF	AECOPD: Caspase-1, oligomeric ASC, and associated cytokines (IL-1β, IL-18) were significantly increased
Di Stefano et al. (35)	2014	COPD	Human bronchial mucosa and BALF	Increase IL-1 and IL-18
Rotta et al. (36)	2013	AECOPD	Mouse macrophage cells, human alveolar macrophages and human lung tissue	NLRP3 inflammasome is not activated in patients with stable COPD
Pauwels et al. (37)	2011	COPD	Mouse lung tissue	Increase NLRP3, caspase-1 and IL-1β
Mortaz et al. (38)	2011	COPD	Human bronchial epithelial cells	CS-induced inflammation occurred independently of IL-1β activation by the NLRP3/caspase-1 axis
Zhang et al. (39)	2018	ALI	Mouse lung tissue, mouse alveolar macrophages	Increase caspase-1 and IL-1β
				Increase NLRP3, caspase-1 and IL-1β
Mehta et al. (40)	2020	Atherosclerosis	Human THP-1 monocytes, macrophages, and foam cells	Activate MyD88/NF-κB pathway and increase NLRP3, caspase-1, IL-1β, and IL-18.
Wu et al. (41)	2018	Atherosclerosis	Mouse aortic tissue, human aortic endothelium cells	Induce ROS production and increase NLRP3, ASC, caspase-1, pro-caspase-1, IL-1β, and IL-18
Yao et al. (42)	2019	Atherosclerosis	Rat vascular smooth muscle cells, rat aortic tissue	Induce ROS production and increase NLRP3
Zheng et al. (43)	2020	Kidney injury	Mouse kidney tissue, human kidney cells	Induce NLRP6 inflammasome activation *via* alpha7 nicotinic acetylcholine receptor
Wu et al. (44)	2020	Bladder dysfunction	Human bladder tissue, human bladder urothelial cells	Induce oxidative stress injury and the activation of NLRP3 inflammasome
Buscetta et al. (45)	2020	Macrophage dysfunction	Human monocyte-derived macrophages and THP-1 cells	Inhibit NLRP3, caspase-1, IL-1β, and IL-18 acting mainly at the transcriptional level, and increase the caspase-1 activity *via* an NLRP3-independent and TLR4-TRIF-caspase-8-dependent pathway
Singh et al. (46)	2019	Podocyte injury	Mouse podocyte cells	Induce ROS production and increase the colocalization of NLRP3 with ASC, caspase-1 activity, and IL-1β production
Zhang et al. (47)	2019	Endothelial barrier dysfunction	Mouse microvascular endothelial cells and mouse coronary arterial endothelium	Increase HMGB1 and enhance cathepsin B-dependent NLRP3 inflammasome activation
Chen et al. (48)	2019	Endothelial barrier dysfunction	Human umbilical vein endothelial cells	Increase caspase-1, NLRP3, and IL-1β
Wang et al. (49)	2019	Endothelial dysfunction	Rat carotid artery tissue, human umbilical vein endothelial cells	Activate ROS/NLRP3 axis
Ye et al. (50)	2019	Oral leukoplakia	Rat oral mucosal epithelium	Reduce expression of the NLRP3 and diminish the secretion of IL-1β and IL-18 maturing by the NLRP3 inflammasome
Han et al. (51)	2017	Ubiquitin-mediated proteasomal processing	Human monocyte THP-1 cells and mouse lung tissue	Decrease NLRP3 protein abundance *via* increased ubiquitin-mediated proteasomal processing

COPD, chronic obstructive pulmonary disease; BALF, bronchoalveolar lavage fluid; NLRP, nucleotide binding oligomerization domain and leucine, rich repeat containing receptor; IL, interleukin; AECOPD, acute exacerbation of chronic obstructive pulmonary disease; AIM, absent in melanoma; TGF, transforming growth factor; ASC, apoptosis associated speck like protein containing a caspase recruitment domain; TLR, Toll like receptor; TRIF, Toll/IL, 1receptor domain containing adaptor inducing interferon, beta; ROS, reactive oxygen species; HMGB1, high mobility group box 1.

*In vivo*, COPD mouse model was established to explore the effect of cigarette smoke on inflammasome (21, 29, 32, 34, 37). In mouse lung tissue, increased expression of NLRP3 was observed after exposure to cigarette smoke (21, 29); moreover, it was also found that the release of downstream inflammation cytokines significantly increased in mouse bronchoalveolar lavage fluid (BALF) samples (21, 29, 34). Cao et al. (29) concluded that activation of NLRP3 inflammasome was related to cigarette smoke-induced ROS production. Nevertheless, an earlier study by Pauwels et al. (37) demonstrated that cigarette smoke-induced inflammation was independent on the activation of NLRP3/caspase-1/IL-1β axis. Moreover, NLRP10 activation might also be involved in the inflammation cytokine release induced by cigarette smoke (32).

Clinical samples from patients were also used to measure whether inflammasome played a role in the pathogenesis of stable COPD (33, 35). Inconsistent with results of studies *in vitro* and *in vivo*, Faner et al. (33) found that the NLRP3 inflammasome was primed, but not activated in the lung tissue of stable COPD patients; besides, both caspase-1 and ASC were mostly in inactive forms. Also, Di Stefano et al. (35) reported that the NLRP3 inflammasome is not activated in bronchial mucosa and BALF of stable COPD patients. However, gene expression analyses revealed that polymorphisms in NLRP1 rs12150220 were associated with COPD disease severity, which suggested the importance of NLRP1 inflammasome fine-tuning in maintaining lung tissue integrity and treating chronic airway inflammation (25). Significant heterogeneity of clinical presentation and disease progression exists in COPD patients, so phenotyping COPD patients is beneficial to identify patient subgroups with unique prognostic or therapeutic characteristics (52). The Spanish guidelines describe four clinical phenotypes for COPD, including chronic bronchitis phenotype, emphysema phenotype, asthma-COPD overlap syndrome phenotype, and non-exacerbator phenotype (53). Interestingly, chronic bronchitis phenotype of COPD is associated with worse respiratory symptoms and higher risk of exacerbations in contrast to other phenotypes (54). However, there have been no studies assessing activation levels of inflammasomes among different phenotypes of COPD patients. Importantly, it seems significant to evaluate whether a higher proportion of activated inflammasomes exists in chronic bronchitis phenotype of COPD patients compared with other phenotypes, as this may contribute a lot to the precision treatment of COPD.

#### Acute Exacerbation of Chronic Obstructive Pulmonary Disease (AECOPD)

Previous studies also proved that the activation of NLRP3 inflammasome was involved in the pathogenesis of AECOPD. Cigarette smoke could enhance the NLRP3 inflammasome activation, and further promote the downstream inflammatory cytokines’ release (including IL-1β and IL-18) in human bronchial epithelial cells (23, 24), and human alveolar epithelial cells (26). Of note, inducing factors of acute exacerbations varied in different studies, such as lipopolysaccharide (23, 26), lipoteichoic acid (23), extracellular heat shock protein 70 (23), and influenza A virus (24) etc. In addition, Rotta et al. (36) verified that there was an upregulation of the NLRP3 in murine macrophage cells and human alveolar macrophages from *Nontypeable Haemophilus Influenzae* infection (NTHi)-induced AECOPD disease model, which imitated the pathogenesis of bacterial exacerbations in COPD. In vivo, animal experiments showed that NLRP3 inflammasome activation existed in AECOPD rat lung tissue and BALF (24).

A growing number of studies demonstrated that there were significant increases of NLRP3 and IL-1β in human peripheral blood mononuclear cells from AECOPD patients (23, 30). Furthermore, potential NLRP3 inflammasome activation accompanied with inflammation cytokine release were also observed in human bronchial tissues (30, 36), serum (30), BALF (30), sputum (33), and plasma (33). Interestingly, Colarusso et al. (28) found that AIM2 inflammasome was also activated in human peripheral blood mononuclear cells derived from AECOPD patients, and could further lead to the release of IL-1α and TGF-β.

#### Cigarette smoke-induced acute lung injury (ALI)

As for smoke inhalation-induced acute lung injury, Zhang et al. (39) reported that cigarette smoke was able to augment the formation of NLRP3 inflammasome, the activation of caspase-1 and IL-1β induced in mouse alveolar macrophages.

### Inflammasome in Cigarette Smoke-Related Cardiovascular Diseases

Recently, there have been studies investigating the role of inflammasome in cigarette smoke-induced atherosclerosis, and these studies mainly focused on NLRP3 inflammasome (40–42, 55). Mehta et al. (55) used human THP-1 monocytes, macrophages and foam cells to represent crucial stages of initiation, progression and development in cigarette smoke-induced atherogenesis, and it was found that cigarette smoke exposure could activate NLRP3 inflammasome in these cells with a stage-specific manner; furthermore, they proved that MyD88/NF-kappa B pathway was an upstream regulator of NLRP3 inflammasome (40). In rat vascular smooth muscle cells and rat aortic tissue, activation of reactive oxygen species (ROS)-NLRP3 inflammasome-C reactive protein (CRP) axis significantly enhanced after nicotine treatment (42). Moreover, nicotine induced ROS-NLRP3-mediated endothelial cell pyroptosis in human aortic endothelial cells (HAECs), which was evidenced by cleavage of caspase-1, production of downstream interleukin IL-1β and IL-18 (41). Importantly, one recent study found that transcriptional and translational expression of NLRP3 inflammasome markers (including caspase-1, pro-IL-1β, IL-1β, pro-IL-18 and IL-18) in mononuclear cells were significantly increased (2 to 7-fold) in smokers with coronary artery disease (CAD) in contrast to non-smokers with CAD (56).

### Inflammasome in Cigarette Smoke-Related Urinary Diseases

Two recent researches concentrated on the change of inflammasome in cigarette smoke-related urinary diseases, including kidney injury and bladder dysfunction (43, 44). Zheng et al. (43) proposed that nicotine induced NLRP6 inflammasome activation *via* alpha7 nicotinic acetylcholine receptor in human kidney cells and mouse kidney tissue. Besides, Wu et al. (44) found that cigarette smoke induced the pyroptosis of urothelial cells through ROS/NLRP3/caspase-1 signaling pathway in bladder dysfunction models.

## Inflammasome in Cigarette Smoke-Related Physiopathological Disorders

Inflammasome might also play an important role in some other physiopathological disorders induced by cigarette smoke, such as macrophage dysfunction (45), endothelial barrier dysfunction (47–49), podocyte injury (46), ubiquitin-mediated proteasomal processing (51), and oral leukoplakia (50) etc.

With regard to macrophage dysfunction, the study by Buscetta et al. (45) manifested that cigarette smoke restrained the expression of NLRP3, caspase-1, IL-1β, and IL-18 acting mainly at the transcriptional level in human monocyte-derived macrophages and THP-1 cells, and increased the caspase-1 activity *via* an NLRP3-independent and Toll-like receptor 4 (TLR4)-Toll/IL-1receptor domain containing adaptor inducing IFN-β (TRIF)-caspase-8-dependent pathway. On the contrary, Rumora et al. (22, 23) found that cigarette smoke was an activated factor of NLRP3 inflammasome in human monocyte-derived macrophages and THP-1 cells.

Endothelial barrier injury has been increasingly considered as an important pathophysiological process in COPD. Chen et al. (48) verified that cigarette smoke could increase the expression of caspase-1, NLRP3, and IL-1β in human umbilical vein endothelial cells. In mouse microvascular endothelial cells and mouse coronary arterial endothelium, nicotine was proved to increase high mobility group box 1(HMGB1) expression, cause NLRP3 inflammasome complex formation and enhance the inflammasome activity as demonstrated by increased cleavage of pro-caspase-1, and IL-1β production (47). In addition, Wang et al. (49) pointed out that cigarette smoke could activate ROS/NLRP3 axis in rat carotid artery tissue and human umbilical vein endothelial cells.

In addition, Singh et al. (46) found that nicotine instigated mouse podocyte cell injury *via* inducing ROS production and activating NLRP3 inflammasome. Han et al. (51) proved that cigarette could decrease NLRP3 protein abundance *via* increased ubiquitin-mediated proteasomal processing. Another interesting finding was that long-term cigarette smoking suppressed NLRP3 inflammasome activation in oral mucosal epithelium and attenuated host defense against Candida albicans in a rat model (50).

Inconsistent conclusions of cigarette smoke on NLRP3 expression among different studies may result from the following reasons. Firstly, Baroja-Mazo et al. (57) reported that the NLRP3 inflammasome particle was released from macrophages after inflammasome activation; thus, the decreased cellular level of NLRP3 protein may be due to the secretion of NLRP3 protein as extracellular oligomeric complexes. Secondly, the preparation method and treated concentration of cigarette smoke extract could not be homogenized among different experiments.

## Therapeutic Strategies to Target Inflammasome in Cigarette Smoke-Related Diseases and Physiopathological Disorders

**Table 2** illustrates therapeutic strategies to target inflammasome in cigarette smoke-induced diseases and physiopathological disorders. Scavenging ROS has become the most widely investigated strategy to inhibit NLRP3 inflammasome activation induced by cigarette smoke. N-Acetyl-L-cysteine (NAC) is an amino-acid derivative of cysteine and a precursor of the antioxidant enzyme glutathione (58). The application of NAC as an antioxidant to diminish ROS generation has been proved in previous studies (59, 60). In clinical practice, NAC has been used as a mucolytic to help clear mucus in patients with respiratory diseases (61). Several studies verified that NAC could decrease ROS generation and further inhibit NLRP3 inflammasome activation, and NAC-mediated disease/disorder involved atherosclerosis (41), bladder damage (44), and podocyte injury (46). Besides NAC, other therapies antagonizing ROS were also explored to modulate inflammasome activation in cigarette smoke-induced diseases. Melatonin (N-acetyl-5-methoxytryptamine) is a neuroendocrine hormone synthesized by tryptophan and serotonin metabolism, and secreted from the pineal gland (62, 63). Melatonin was found to play an important role in relieving oxidative stress injury (64, 65). One study by Wang et al. (49) illustrated that melatonin inhibited ROS production, NLRP3 inflammasome activation and pyroptosis in cigarette smoke-treated endothelial cells. Another effect of melatonin on NLRP3 inflammasome activation was to suppress endoplasmic reticulum stress and mitochondrial dysfunction in COPD (21). Inhibitors of transient receptor potential protein (TRP) ion channels (TRPA1 and TRPV1) might also attenuate NLRP3 inflammasome activation by reducing oxidative stress and blocking Ca^2+^ influx (27). Moreover, rosmarinic acid and lipoxin receptor agonist (BML-111) were also capable of mitigating ROS production and restraining NLRP3 inflammasome activation in cigarette smoke-induced diseases (29, 42).

**Table 2:** Therapeutic strategies to target inflammasome in cigarette smoke-induced diseases and physiopathological disorders.

Reference	Year	Disease/disorder	Therapy	Mechanism
Wu et al. (41)	2018	Atherosclerosis	NAC	Inhibit ROS
Wu et al. (44)	2020	Bladder damage	NAC	Inhibit ROS
Singh et al. (46)	2019	Podocyte injury	NAC	Scavenge ROS
Wang et al. (49)	2019	Endothelial dysfunction	Melatonin	Inhibit ROS/NLRP3 axis
Mahalanobish et al. (21)	2020	COPD	Melatonin	Suppress endoplasmic reticulum stress, mitochondrial dysfunction and further inhibit NLRP3 inflammasome activation
Wang et al. (27)	2019	COPD	Transient receptor potential protein ion channel inhibitors	Reduce oxidative stress, block Ca^2+^ influx, and inhibit NLRP3 inflammasome activation
Yao et al. (42)	2019	Arteriosclerosis	Rosmarinic acid	Inhibit the ROS-NLRP3 inflammasome-CRP axis
Cao et al. (29)	2018	COPD	Lipoxin receptor agonist	Inhibit ROS production and prevent NLRP3 inflammasome activation
Ji et al. (24)	2020	AECOPD	Shufeng Jiedu Capsule (SFJDC), oseltamivir	Inhibit NLRP3 inflammasome activation
Zhang et al. (47)	2019	Endothelial barrier dysfunction	Blockade of HMGB1	Inhibit NLRP3 inflammasome activation
Chen et al. (48)	2019	Endothelial barrier dysfunction	Mitoquinone	Inhibit NLRP3 inflammasome activation
Zhang et al. (39)	2018	ALI	Suppressor of cytokine signaling-1 (SOCS-1)	Dampen the formation of NLRP3 inflammasome and the activation of caspase-1 and IL-1β
Singh et al. (31)	2018	COPD	Poly-unsaturated fatty acids (PUFA)	Inhibit membrane recruitment of NLRP10 and NLRP12

NAC, N, Acetyl, L, cysteine; ROS, reactive oxygen species; NLRP, nucleotide binding oligomerization domain and leucine, rich repeat containing receptor; COPD, chronic obstructive pulmonary disease; CRP, C reactive protein; AECOPD, acute exacerbation of chronic obstructive pulmonary disease; HMGB1, high mobility group box 1; ALI, acute lung injury; IL, interleukin.

There were also some other strategies to target NLRP3 inflammasome in cigarette smoke-related diseases. Concerning endothelial barrier dysfunction induced by cigarette smoke, Zhang et al. (47) found that blockade of HMGB1 could inhibit NLRP3 inflammasome activation; similarly, mitoquinone was also able to diminish NLRP3 inflammasome activation in cigarette smoke-induced endothelial barrier dysfunction (48). With respect to cigarette smoke induced-ALI, suppressor of cytokine signaling-1 (SOCS-1) might dampen the formation of NLRP3 inflammasome and the activation of caspase-1 and IL-1β (39). Futhermore, Ji et al. (24) pointed out that Shufeng Jiedu Capsule (SFJDC) and oseltamivir significantly decreased NLRP3 inflammasome activation in influenza virus A-induced AECOPD disease models.

Targeting NLRP10 and NLRP12 inflammasome, poly-unsaturated fatty acids (PUFA) was capable of rescuing A549 cells from cigarette smoke extract (CSE)-mediated membrane recruitment of NLRP10 and NLRP12, and also from inflammatory responses (31). Finally, **Supplemental Figure 1** summarized effects of cigarette smoke on NLRP3 inflammasome, and potential therapeutic strategies.

## Summary

Collectively, emerging evidence demonstrates that inflammasomes (NLRP3, NLRP6, NLRP12 and AIM2) are involved in the pathogenesis of several cigarette smoke-related diseases (including COPD, ALI, atherosclerosis, kidney injury, bladder dysfunction, and oral leukoplakia) and physiopathological disorders (macrophage dysfunction, endothelial barrier dysfunction, podocyte injury, and ubiquitin-mediated proteasomal processing). MyD88/NF-κB, HMGB1, production of ROS, endoplasmic reticulum stress and mitochondrial dysfunction, and Ca^2+^ influx are potentially involved in cigarette smoke induced-inflammasome activation. Strategies targeting ROS/NLRP3 inflammasome axis are most widely investigated and show potential therapeutic effects. Although this review reveals the potential relationship between cigarette smoke and inflammasome, more studies may be still needed to further confirm more detailed mechanisms so as to provide effective alternatives for treating cigarette smoke-related diseases.

## Author Contributions

All authors contributed to the article and approved the submitted version.

## Funding

This work was supported by National Natural Science Foundation of China (No. 81873410, No. 81800043, and No. 82070049), and National Natural Science Foundation of Hunan (No. 2020JJ5818).

## Conflict of Interest

The authors declare that the research was conducted in the absence of any commercial or financial relationships that could be construed as a potential conflict of interest.

## Supplementary Material

Supplementary Figure 1 Effect of cigarette smoke on NLRP3 inflammasome, and potential therapeutic strategies. HMGB, high mobility group box 1; NAC, N-Acetyl-L-cysteine; ROS, reactive oxygen species; TRP, transient receptor potential protein; ER, endoplasmic reticulum; NLRP, Nucleotide binding oligomerization domain and leucine-rich repeat containing receptor.
