# Supplementary figures and images for: Roles of Inflammasome in Cigarette Smoke-Related Diseases and Physiopathological Disorders: Mechanisms and Therapeutic Opportunities
Source: Front Immunol. 2021 Jul 21;12:720049. doi: 10.3389/fimmu.2021.720049 (PMC8334727; doi:10.3389/fimmu.2021.720049)

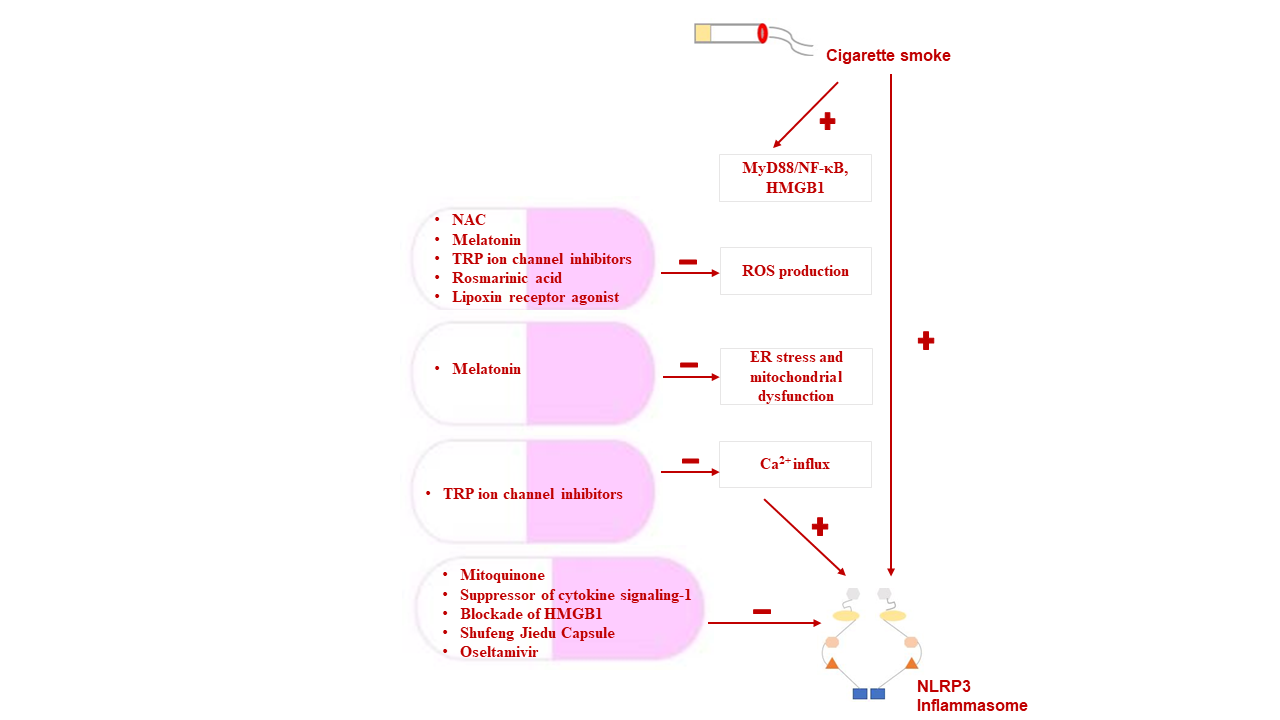

Supplement: Supplementary Figure 1 — Effect of cigarette smoke on NLRP3 inflammasome, and potential therapeutic strategies. HMGB, high mobility group box 1; NAC, N-Acetyl-L-cysteine; ROS, reactive oxygen species; TRP, transient receptor potential protein; ER, endoplasmic reticulum; NLRP, Nucleotide binding oligomerization domain and leucine-rich repeat containing receptor. [file Image_1.tif]
